# Genome-Wide Identification of the TRAF Gene Family in Common Carp (*Cyprinus carpio*) and Analysis of Their Expression in Response to CyHV-3 Challenge

**DOI:** 10.3390/ani16182859

**Published:** 2026-09-11

**Authors:** Yaxin Di, Xiaona Jiang, Chitao Li, Xuesong Hu, Yanlong Ge, Xiaodan Shi, Xinyu Zhao, Zhiying Jia

**Affiliations:** 1 Heilongjiang River Fisheries Research Institute, Chinese Academy of Fishery Sciences, Harbin 150076, China; diyaxin@hrfri.ac.cn (Y.D.);; 2 Key Laboratory of Freshwater Aquatic Biotechnology and Breeding, Ministry of Agriculture and Rural Affairs, Harbin 150070, China

**Keywords:** common carp, TRAF, CyHV-3, gene expression, phylogenetic analysis

## Abstract

Tumor necrosis factor receptor-associated factors (TRAFs) are key signal transduction molecules in innate immunity and are involved in multiple immune pathways, such as TNF receptor and Toll-like receptor signaling. In this study, 16 *CcTRAFs* were identified from the common carp genome, assigned to six subfamilies (*TRAF1*/*2*/*3*/*4*/*6*/*7*), with no *TRAF5* found. Significant paralogous expansion was observed in the *TRAF2*/*4* subfamilies. All CcTRAF proteins were predicted to be unstable and hydrophilic by ProtParam analysis; most contain conserved RING, TRAF and MATH domains, whereas CcTRAF7 carries seven WD40 domains. Gene structure and conserved motif analysis revealed high conservation of exon numbers and motif compositions within each subfamily, while the TRAF7 subfamily displayed distinct characteristics. Collinearity analysis further indicated that segmental duplication promotes *CcTRAF* family expansion. qRT-PCR showed that *CcTRAF* genes have tissue-specific expression patterns and are preferentially expressed in the liver, spleen, and kidney. Under CyHV-3 infection, the expression levels of *CcTRAF3*/6/*7* in these tissues first increased and then decreased, peaking at 48 h post-challenge. In summary, *CcTRAF* genes, especially *CcTRAF3*/6/*7*, play important roles in the immune response of common carp against CyHV-3. This study provides a molecular basis for understanding innate immune regulation in teleost fish and the functional roles of TRAF genes.

## Simple Summary

Common carp (*Cyprinus carpio*) is one of the most economically valuable freshwater fish worldwide. Cyprinid herpesvirus 3 (CyHV-3) infection causes massive mortality and huge economic losses in carp aquaculture. Tumor necrosis factor receptor-associated factors (TRAFs) are essential signal molecules regulating innate immune and antiviral responses in teleosts. Nevertheless, the immune functions of *TRAF* genes in common carp against CyHV-3 remain poorly understood. In this work, we identified 16 *CcTRAF* genes in the common carp genome and explored their tissue distribution and expression responses upon viral challenge. *CcTRAF* genes are preferentially expressed in main immune organs. Under CyHV-3 challenge, *CcTRAF3*, *CcTRAF6* and *CcTRAF7* exhibited significant transcriptional changes and reached expression peaks at 48 h post-challenge. Our results indicate that *CcTRAF* genes participate in carp antiviral immunity. This study provides fundamental clues for understanding TRAF-mediated immune defense in common carp.

## 1. Introduction

The tumor necrosis factor receptor-associated factors (*TRAFs*) family is a group of important immune signal transduction molecules. Since their discovery in the 1990s, TRAFs have been confirmed to be the core regulatory factors of both innate and adaptive immune signaling pathways [1]. Members of this family are widely involved in the signal transmission of Toll-like receptors, NOD-like receptors, RIG-I-like receptors, C-type lectin receptors, T-cell receptors, the tumor necrosis factor receptor superfamily, and various interleukin receptors and interferon receptors, playing a crucial role in many physiological and pathological processes such as anti-infection, inflammatory response, cell apoptosis, proliferation and differentiation, and tumor occurrence in the body [2]. TRAF proteins were initially identified in mammals and subsequently discovered in various model organisms, including nematodes (*Caenorhabditis elegans*) and fruit flies (*Drosophila melanogaster*), indicating that this family is highly conserved throughout evolution [3,4,5]. To date, 7 members of the *TRAF* family have been identified in mammals, including 6 classical members (*TRAF1*-*TRAF6*) and 1 non-classical member, *TRAF7* [6]. Except for TRAF7, most TRAF proteins contain the TRAF domain at the C-terminal. This domain consists of approximately 230 amino acid residues and is an important functional module that mediates protein interactions. It mainly comprises two subdomains: the coiled-coil conformational TRAF-N region and the β-sheet-rich TRAF-C (also known as MATH) domain [7]. In contrast, the C-terminal of *TRAF7* does not possess the MATH domain but is instead replaced by 7 WD40 repeat sequences, which can perform similar functions as the MATH domain [8,9]. Apart from *TRAF1*, the N-terminal of other TRAF proteins all contain conserved RING finger and zinc finger domains [8]. Among them, the RING finger domain confers its E3 ubiquitin ligase activity, regulating the activation of the downstream NF-κB signaling pathway; the zinc finger domain mediates protein interactions and DNA binding, participating in the activation of the NF-κB and JNK pathways [10].

Like mammals, teleost fish also possess TRAFs, and TRAF family members exhibit functional differentiation in the antiviral immune response. For instance, red-spotted grouper (*Epinephelus akaara*) TRAF2 exerts antiviral functions by activating the NF-κB and JNK pathways [11]. In yellow croaker (*Larimichthys crocea*), TRAF3 participates in the TLR signaling pathway to regulate antiviral immunity [12], whereas TRAF4 negatively affects the antiviral immune response by promoting viral replication [13]. Similarly, grouper TRAF5 enhances SGIV replication while significantly activating IFN-β and NF-κB promoter activity, suggesting that the virus may hijack the TRAF5-mediated signaling pathway to facilitate immune evasion [14]. Black carp MAVS transmits antiviral signals by recruiting TRAF6 to regulate the host antiviral immune response [15]. Unlike most TRAF family members, yellow croaker TRAF7 features a C-terminal region containing seven WD40 repeat motifs instead of a canonical MATH domain, and inhibits SVCV replication via the NF-κB and IRF3/7 signaling pathways [16]. Research on *TRAF1* in the antiviral immune response of teleost fish remains limited, and *TRAF1* orthologs appear to be absent in certain species. Collectively, these findings indicate that the TRAF family contributes to the antiviral immune response in teleosts and plays an essential role in host defense against pathogen infection. Current knowledge of the TRAF family in common carp is restricted to a small subset of family members. For instance, common carp *TRAF3* has been shown to participate in the RIG-I antiviral signaling pathway [17], whereas common carp *TRAF6* exists as two paralogous copies and participates in TLR-mediated antiviral immune regulation [17,18]. However, comprehensive identification and functional characterization of other *TRAF* family members in common carp remain lacking, and systematic evolutionary analyses, gene structural architecture, and dynamic expression profiling during CyHV-3 challenge have not yet been reported.

The common carp is the freshwater fish species with the greatest variety, widest distribution, and longest history of aquaculture, and it is also an important economic aquatic species in China. Koi herpesvirus disease (KHVD), caused by CyHV-3, has spread worldwide, causing significant economic losses to the carp aquaculture industry [19]. Therefore, a deeper understanding of the immune response mechanisms in carp is crucial for controlling this disease. In this study, we systematically identified the *TRAF* gene family in the common carp genome using bioinformatics approaches, analyzed their conserved domains, clarified the number of family members, structural characteristics, evolutionary relationships, and tissue expression patterns, and further investigated changes in the expression of *TRAF* family members under CyHV-3 challenge. These findings will help elucidate the role of the *TRAF* family in the innate immune response of common carp to CyHV-3 challenge.

## 2. Materials and Methods

### 2.1. Identification of CcTRAF Gene Family Members

To identify members of the *TRAF* gene family in common carp, the chromosome-level common carp reference genome assembly GCF_018340385.1 (ASM1834038v1) was retrieved from the NCBI RefSeq database (accessed on 12 April 2026). Protein sequences from *Scophthalmus maximus*, *Cynoglossus semilaevis*, *Paralichthys olivaceus*, *Oreochromis niloticus*, *Takifugu rubripes*, *Gasterosteus aculeatus*, *Oryzias latipes*, *Lepisosteus oculatus*, *Xiphophorus maculatus*, *Danio rerio*, *Mus musculus*, and *Homo sapiens* were used as query sequences for Blastp searches against the NCBI protein database to retrieve TRAF homologs in common carp. Additionally, hidden Markov models (HMMs) for the zf-TRAF (Pfam accession: PF02176) and MATH (Pfam accession: PF00917) domains were downloaded from the Pfam database (https://www.ebi.ac.uk/interpro/, accessed on 12 April 2026) and used with TBtools (v1.098769) to search the common carp proteome.

Candidate TRAF proteins obtained from Blastp and HMM searches were further filtered with explicit criteria: sequences were retained only if they contained at least one typical zf-TRAF or MATH conserved domain; partial, fragmented sequences lacking core TRAF-related domains were discarded. The combination of these two approaches was employed to identify and confirm the *TRAF* gene family members in common carp. Further validation of domain architecture for the identified genes was performed using the SMART online tool (https://smart.embl.de/, accessed on 12 April 2026). Physicochemical properties of the proteins encoded by the *CcTRAF* genes were analyzed using the ExPASy ProtParam tool (https://web.expasy.org/protparam/, accessed on 12 April 2026).

### 2.2. Phylogenetic Tree, Conserved Domain and Protein Structure Analysis of CcTRAFs

Amino acid sequences of TRAF proteins used for phylogenetic reconstruction are listed in Supplementary Table S1, including sequences from *Scophthalmus maximus*, *Cynoglossus semilaevis*, *Paralichthys olivaceus*, Oreochromis niloticus, *Takifugu rubripes*, *Gasterosteus aculeatus*, *Oryzias latipes*, *Lepisosteus oculatus*, *Xiphophorus maculatus*, *Danio rerio*, *Mus musculus*, *Homo sapiens*, and *Cyprinus carpio*. Multiple sequence alignment was conducted using ClustalW integrated within MEGA11 (v11.0) software with default protein alignment parameters; gapped columns originating from poorly aligned terminal regions were manually removed before tree inference. A phylogenetic tree was constructed using the neighbor-joining (NJ) distance-based method, with 1000 bootstrap replicates to evaluate branch reliability, implemented in MEGA11.

The conserved domains of the CcTRAF proteins were identified using the online tools SMART (https://smart.embl.de/, accessed on 12 April 2026) and Pfam, and visualized using IBS 6.0. The secondary structures of the CcTRAF proteins were analyzed using the NPS@ server (https://npsa-prabi.ibcp.fr/, accessed on 12 April 2026), and their tertiary structures were predicted using SWISS-MODEL (https://swissmodel.expasy.org/interactive, accessed on 12 April 2026).

### 2.3. Gene Structure, Chromosomal Localization, and Synteny Analysis of CcTRAFs

The exon-intron structures of the *CcTRAF* genes were identified using the Gene Structure Display Server (GSDS) 2.0. Genome data for common carp (*Cyprinus carpio*, assembly GCF_018340385.1), zebrafish (*Danio rerio*, GCF_000002035.6), and human (*Homo sapiens*, GCF_000001405.40) were retrieved from the NCBI Genome database. Chromosomal localization maps of *CcTRAF* gene family members were generated using TBtools. Synteny analysis within *CcTRAFs* and between common carp and zebrafish or human was performed using TBtools (v11.0).

### 2.4. Fish Rearing and Viral Challenge

Common carp (*Cyprinus carpio*) with an average body weight of 8–12 g were obtained from Kuandian Experimental Station, Heilongjiang Fisheries Research Institute, Chinese Academy of Fishery Sciences. Prior to challenge, gill and head kidney tissues from randomly selected fish were examined by PCR with TK- and Sph-specific primers. No target amplicon of CyHV-3 was detected, confirming that experimental fish were CyHV 3 negative. In total, 40 fish were randomly distributed into four independent tanks with an actual water volume of 50 L, including three biological replicate challenge tanks and one control tank, with 10 fish per tank and a stocking density of 2 kg/m^3^. Fish were acclimated for 14 d under a 12 h light/12 h dark photoperiod. Water temperature was maintained at 24 ± 1 °C and pH at 7.0 ± 0.2 with continuous strong aeration. Fish were fed twice daily during acclimation, and feeding was terminated after viral challenge. Fish were transferred by container instead of a fishing net to prevent damage to epidermal mucus.

CyHV 3 immersion challenge was performed according to the previously reported method of Jia et al. [20]. The viral inoculum was prepared as tissue homogenate from internal organs of carp showing typical CyHV 3 clinical signs. The presence of CyHV 3 in diseased fish tissues was verified by PCR targeting TK and Sph genes following the industry standard SC/T 7212.1-2011 [20]. The viral load of the homogenate was 1.5 × 10^7^ copies per mg of tissue, as determined by qPCR targeting the Sph gene. For immersion challenge, 1 mL of tissue homogenate was added into each challenge tank, while the control tank received an equal volume of sterile PBS. The control group was only used for clinical symptom observation without sequential tissue sampling.

Each independent tank was defined as one experimental unit (biological replicate). At 0, 24, 48, 72, 96 and 120 h post-challenge, a single fish was randomly sampled from each tank as a representative individual of that tank. Thus, three independent tanks produced three biological replicates at each time point for qPCR analysis. Fish were anesthetized with 100 mg/L MS-222. Tissues including liver, head kidney, kidney, spleen, intestine, heart, gill and brain were dissected, snap-frozen in liquid nitrogen and stored at −80 °C for subsequent RNA extraction. Post-challenge PCR amplification of the TK and Sph genes verified that all fish sampled at each time point were successfully infected with CyHV-3, indicating that the challenge model was effective and reliable for subsequent expression analysis.

In this study, all fish handling and animal experiments were conducted strictly in accordance with the Guidelines for the Care and Use of Laboratory Animals of the Heilongjiang River Fisheries Research Institute, Chinese Academy of Fishery Sciences (Harbin, China), and were approved by the Institutional Animal Care and Use Committee (IACUC) of the institute (approval code: 2019-03-15, approval date: 15 March 2019).

### 2.5. Total RNA Extraction and qRT-PCR Detection

Total RNA was extracted from virus-infected frozen tissues using an RNeasy Mini Kit (QIAGEN, Frankfurt, Germany) in accordance with the manufacturer’s instructions, including tissue homogenization and lysis. The integrity and quality of RNA were assessed via 1.0% agarose gel electrophoresis, while its purity was quantified using ultraviolet (UV) spectrophotometric analysis. Qualified RNA samples with OD_260_:OD_280_ ratios ranging from 1.8 to 2.0 were used for subsequent cDNA synthesis. cDNA was synthesized with the PrimeScript FAST RT Reagent Kit with gDNA Eraser (TAKARA, Dalian, China).

Quantitative real-time PCR (qPCR) was carried out using TB Green Fast Premix Ex Taq II (TAKARA, Dalian, China) on an Agilent Technologies Stratagene Mx3005P real-time thermal cycler (Agilent Technologies, Santa Clara, CA, USA). All primer pairs were designed for *CcTRAF* genes, and primer specificity was strictly validated. Before qPCR experiments, in silico sequence alignment was performed for each primer set against all identified *CcTRAF* family coding sequences to prevent cross-amplification among highly homologous paralogs. Melt-curve analysis was conducted after each qPCR reaction. A single sharp melting peak was observed for every primer pair, verifying specific amplification of target fragments and excluding non-specific products and paralog cross-amplification. The primer sequences, corresponding gene accession numbers and related information are listed in Table 1. *β-Actin* was selected as the internal reference gene. The relative expression levels of candidate genes were calculated using the 2^−ΔΔCt^ method.

### 2.6. Statistical Data Analysis

The relative expression levels of candidate genes were calculated using the 2^−ΔΔCt^ method. All statistical analyses were performed based on tank-level biological replicates. Experimental data are presented as the mean ± standard deviation (mean ± SD). Before statistical comparison, the normality of data distribution and homogeneity of variance were examined. One-way analysis of variance (ANOVA) followed by Tukey’s multiple-comparison test was conducted using GraphPad Prism 8.0 software. The significance threshold was set at *p* < 0.05.

## 3. Results

### 3.1. Identification and Physicochemical Properties of CcTRAF Family

A total of 16 TRAF genes were identified in the common carp genome, named *CcTRAF1*, *CcTRAF2aa*, *CcTRAF2aa1*, *CcTRAF2ab*, *CcTRAF2ab1*, *CcTRAF2b*, *CcTRAF2ba*, *CcTRAF3a*, *CcTRAF3b*, *CcTRAF4a*, *CcTRAF4aa*, *CcTRAF4b*, *CcTRAF6a*, *CcTRAF6b*, *CcTRAF7*, and *CcTRAF7a*. Physicochemical analysis of the CcTRAF family members revealed that the encoded proteins ranged from 470 to 725 amino acids in length, with molecular weights ranging from 54,395.36 to 81,142.81 Da and isoelectric points ranging from 6.29 to 8.55 (Table 2). The protein instability index was greater than 40.00 for all members, indicating that these proteins were predicted to be unstable according to the ProtParam instability index; the aliphatic index ranged from 63.26 to 84.39; and the average hydrophobicity index was less than zero, suggesting that all of them were hydrophilic proteins.

### 3.2. Structural Characterization of CcTRAF Proteins

The results of protein secondary structure prediction are shown in Table S2. All members of the CcTRAF family contain α-helices, β-turns, extended chains, and random coil structures. Differences in predicted secondary-structure composition are observed among TRAF subfamilies: the TRAF1/2 subfamily exhibits the highest α-helical content, followed by random coils; in contrast, the TRAF3/4/6 subfamily displays a higher proportion of random coils, with α-helices as the second most abundant secondary structure. Meanwhile, the TRAF7 subfamily shows a lower proportion of α-helices and a higher proportion of extended strands, displaying structural characteristics entirely distinct from those of other TRAF members, which may imply functional evolutionary specialization. Overall, the CcTRAF family retains core structural conservation while undergoing structural diversification to accommodate distinct immune regulatory functions. The predicted tertiary structures show high similarity among members of the same subfamily, with obvious differences observed between different subfamilies (Figure S1). Model quality assessment indicates that the homology models for the 16 CcTRAF family members exhibit GMQE values ranging from 0.73 to 0.92, with template-derived sequence identity ranging from 74.20% to 98.94%. The obtained homology models are putative structural predictions, evaluated based on GMQE, sequence identity and coverage metrics (Table S3).

### 3.3. Phylogenetic Analysis of CcTRAFs

**Figure 1:**
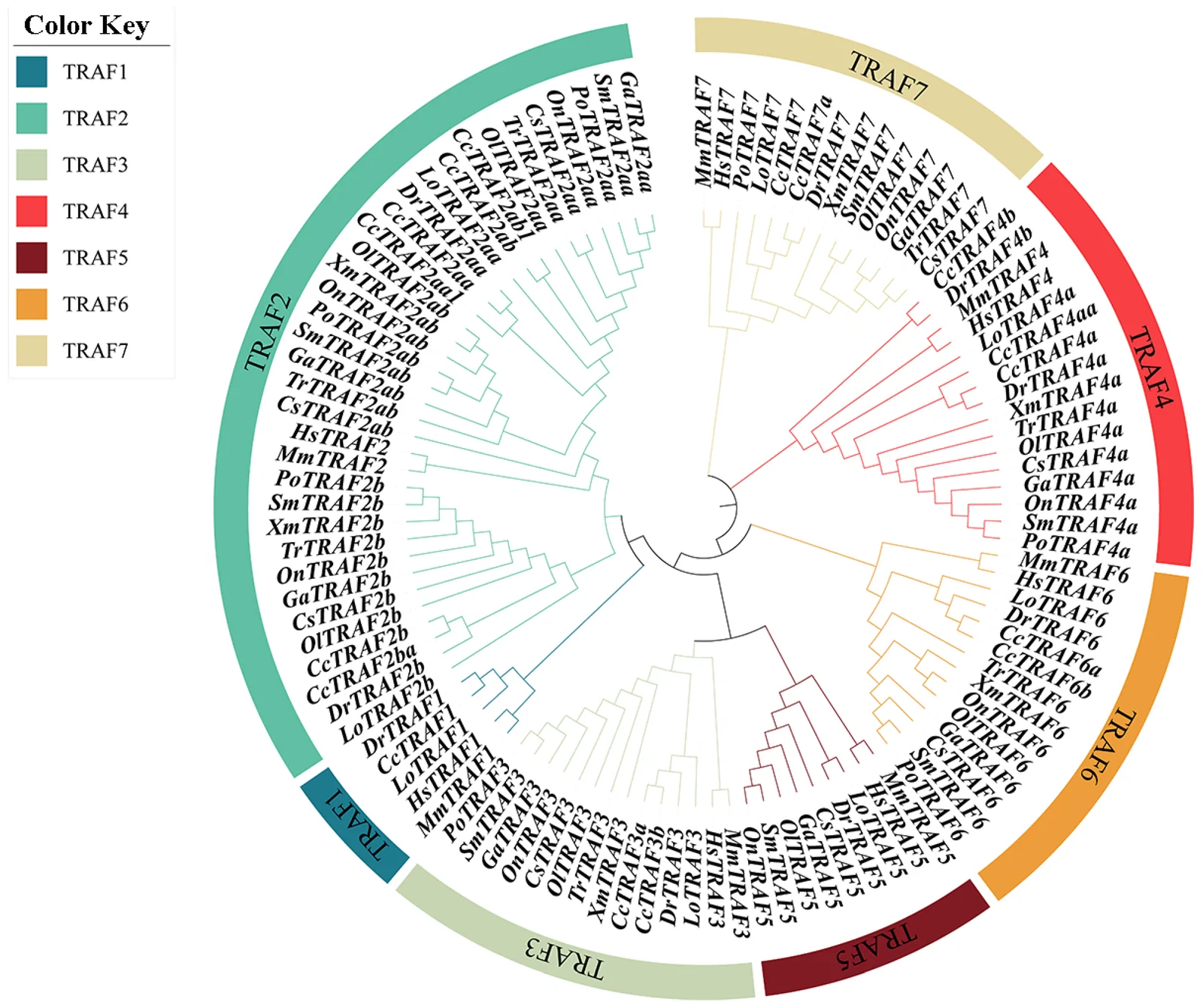
Phylogenetic relationship of *TRAF* genes from 13 different vertebrate species. Sm: *Scophthalmus maximus*, Cs: *Cynoglossus semilaevis*, Po: *Paralichthys olivaceus*, On: Oreochromis niloticus, Tr: *Takifugu rubripes*, Ga: *Gasterosteus aculeatus*, Ol: *Oryzias latipes*, Lo: *Lepisosteus oculatus*, Xm: *Xiphophorus maculatus*, Dr: *Danio rerio*, Mm: *Mus musculus*, Hs: *Homo sapiens*, Cc: *Cyprinus carpio*.

To investigate the phylogenetic relationships of the *CcTRAFs*, this study constructed a phylogenetic tree using TRAF sequences from common carp and other species. As shown in Figure 1, a total of 107 *TRAF* genes from 13 species were classified into seven subfamilies. Subfamily I contained five *TRAF1* genes, subfamily II included 35 *TRAF2* genes, subfamily III had 14 *TRAF3* genes, subfamily IV comprised 16 *TRAF4* genes, subfamily V consisted of nine *TRAF5* genes, subfamily VI contained 14 *TRAF6* genes, and subfamily VII included 14 *TRAF7* genes. The clustering of species within each subfamily followed vertebrate evolutionary patterns, with cyprinid fish *TRAF* members grouping together first, reflecting sequence homology among closely related species. Notably, subfamilies *TRAF2* and *TRAF4* showed paralogous expansion, whereas common carp lacked the *TRAF5* gene, indicating species-specific evolutionary characteristics. These results demonstrate that *TRAF* genes within each subfamily formed highly conserved phylogenetic clades across species, highlighting conservation at the subfamily level.

### 3.4. Gene Structure Features of CcTRAFs

**Figure 2:**
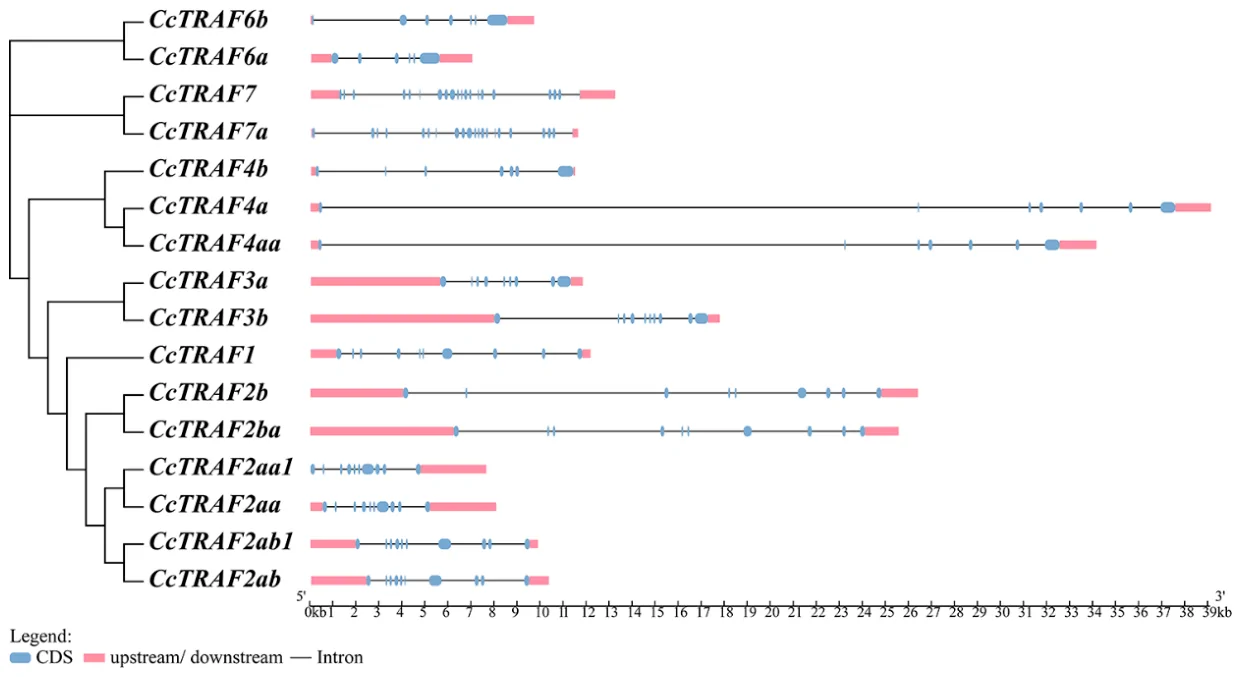
The structure of *CcTRAF* genes.

To understand the gene structure of the *CcTRAFs*, we performed gene structure analysis using the GSDS online tool (Figure 2). The results showed that subfamily VII contained the highest number of exons, with 20, while subfamily IV had the fewest, with only 7 exons. Interestingly, each subfamily exhibits a relatively consistent number of exons, further indicating high conservation within these subfamilies.

### 3.5. Conserved Domains, Motif Patterns and Chromosomal Distribution of CcTRAFs

**Figure 3:**
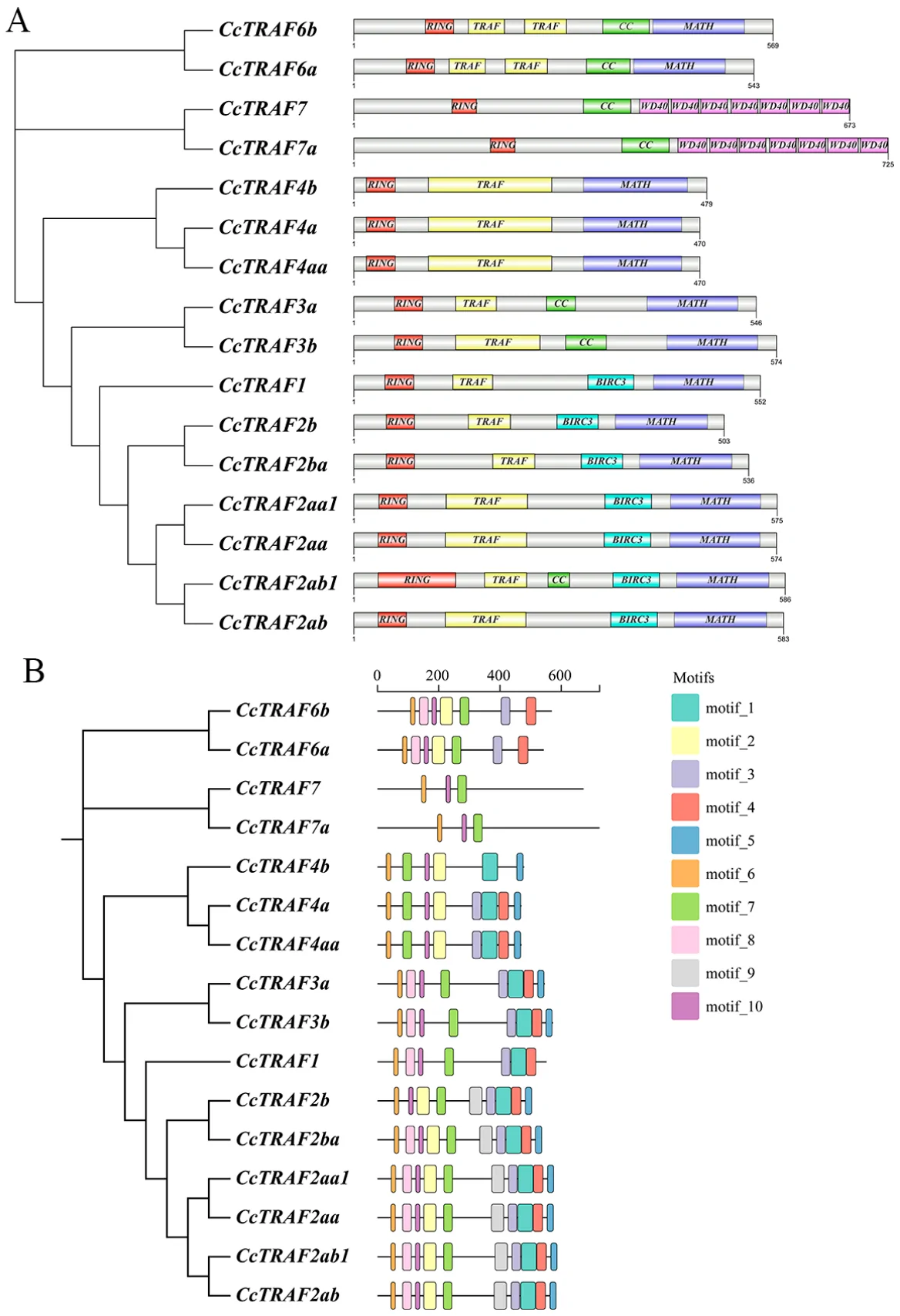
Conserved structural domains (**A**) and conserved motifs (**B**) of encoded proteins of the *CcTRAF* gene family members.

**Figure 4:**
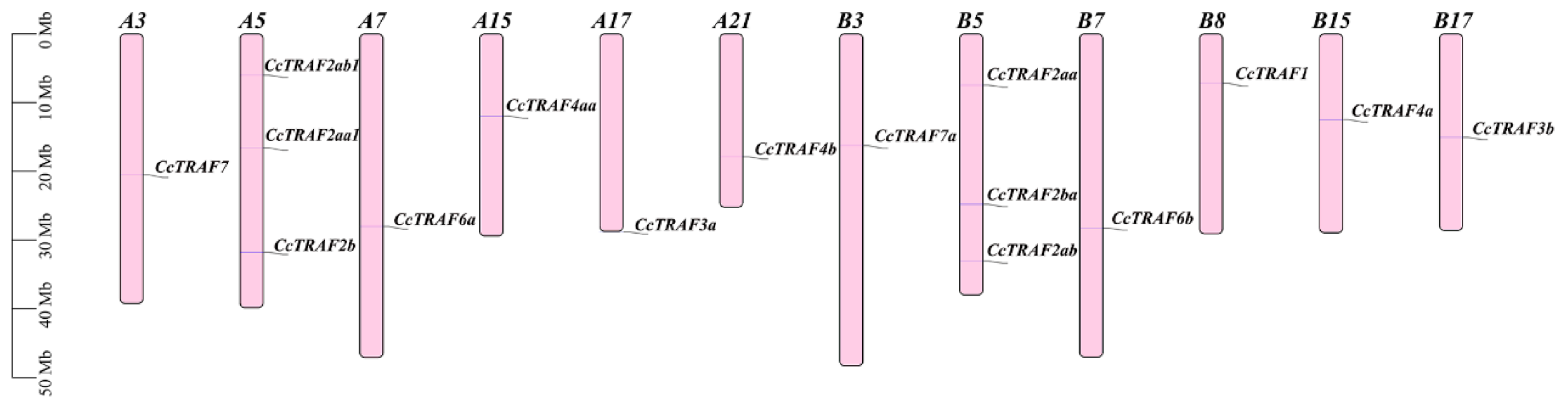
Chromosomal localization map of the *CcTRAF* gene family members.

To characterize the conservation of CcTRAFs among subfamilies, we further conducted analyses of conserved domains and motifs. As shown in Figure 3A, all CcTRAF proteins except CcTRAF7 contain the conserved RING, TRAF, and MATH domains. Notably, CcTRAF7 possesses a canonical set of seven WD40 repeats. Interestingly, each subfamily possesses its own distinctive set of conserved domains. Subfamilies I and II share highly similar domain architectures, both containing a BIRC3 domain. In contrast, subfamily IV contains three canonical TRAF domains, whereas subfamily VI includes two canonical TRAF domains and one coiled-coil domain. Further analysis of conserved motifs in the *CcTRAFs* revealed ten motifs across the family (Figure 3B). Except for CcTRAF7, the motifs present in other CcTRAF proteins are largely similar. The motif composition of CcTRAF7 is distinctly different from that of other CcTRAF proteins, indicating that CcTRAF7 is unique within the CcTRAF family, a finding consistent with the domain conservation analysis. These results suggest that each subfamily performs distinct functional roles in common carp. Chromosomal localization analysis showed that *CcTRAFs* were evenly distributed across different chromosomes, located on chromosomes A3, A5, A7, A15, A17, A21, B3, B5, B7, B8, B15, and B17 (Figure 4).

### 3.6. Synteny Analysis of CcTRAF Genes

**Figure 5:**
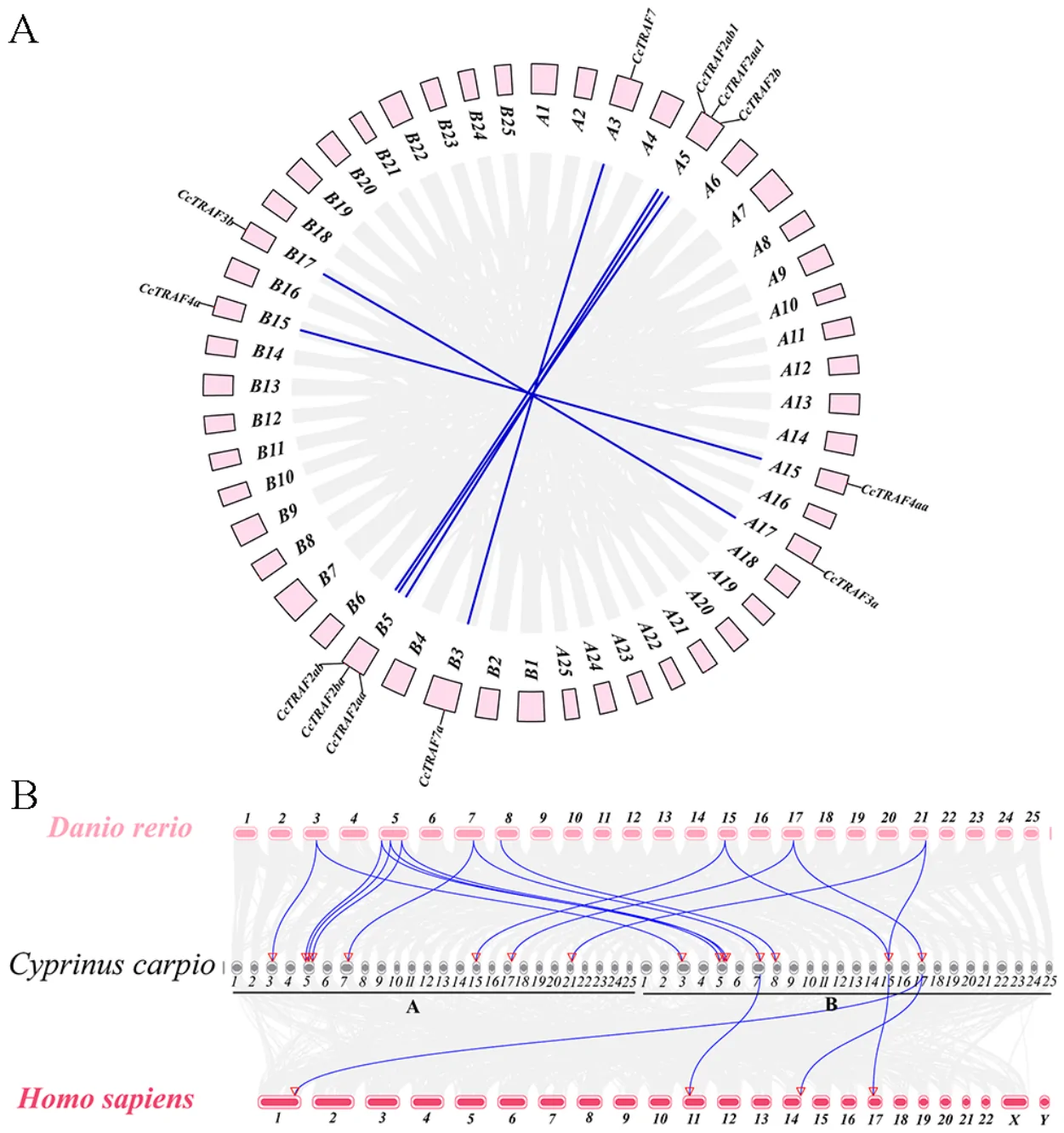
Collinearity analysis of *CcTRAF* gene family members. (**A**) Intraspecific collinearity of CcTRAF genes in common carp (*Cyprinus carpio*). The plot shows the chromosomal distribution and duplicated *CcTRAF* gene pairs within the common carp genome. (**B**) Interspecific collinearity of *TRAF* genes among zebrafish (*Danio rerio*), common carp (*Cyprinus carpio*), and human (*Homo sapiens*). Blue lines indicate collinear *TRAF* gene pairs, while grey lines represent collinear relationships of other genomic genes unrelated to the *TRAF* family. Chromosome numbering is species-specific and independent across the three species.

To explore the evolutionary expansion mechanism of the *CcTRAF* gene family in common carp, intraspecific and interspecific synteny analyses were performed on all *CcTRAF* members. Intraspecific synteny results (Figure 5A) demonstrate robust collinearity across multiple *CcTRAF* genes, demonstrating segmental duplication as the primary force driving *CcTRAF* family expansion. Multiple paralogs belonging to the *CcTRAF2* subfamily, including *CcTRAF2aa*, *CcTRAF2ab* and *CcTRAF2b*, share collinear genomic regions, which verifies lineage-specific duplication events that expanded this subfamily during common carp evolution. Other subfamily genes *CcTRAF3*, *CcTRAF4*, *CcTRAF6* and *CcTRAF7* also retain syntenic blocks to different extents, providing additional evidence for pervasive segmental duplication throughout the *CcTRAF* family. Interspecific synteny comparisons uncovered highly conserved chromosomal fragments between common carp TRAF genes and their zebrafish orthologs, with paired homologous segments consistent with the teleost-specific whole-genome duplication (Ts3R) event in cyprinids. By contrast, weak syntenic conservation was observed between fish and human TRAF loci, indicative of large-scale chromosomal rearrangements following vertebrate lineage divergence (Figure 5B).

### 3.7. Tissue Expression Profiles of CcTRAFs

**Figure 6:**
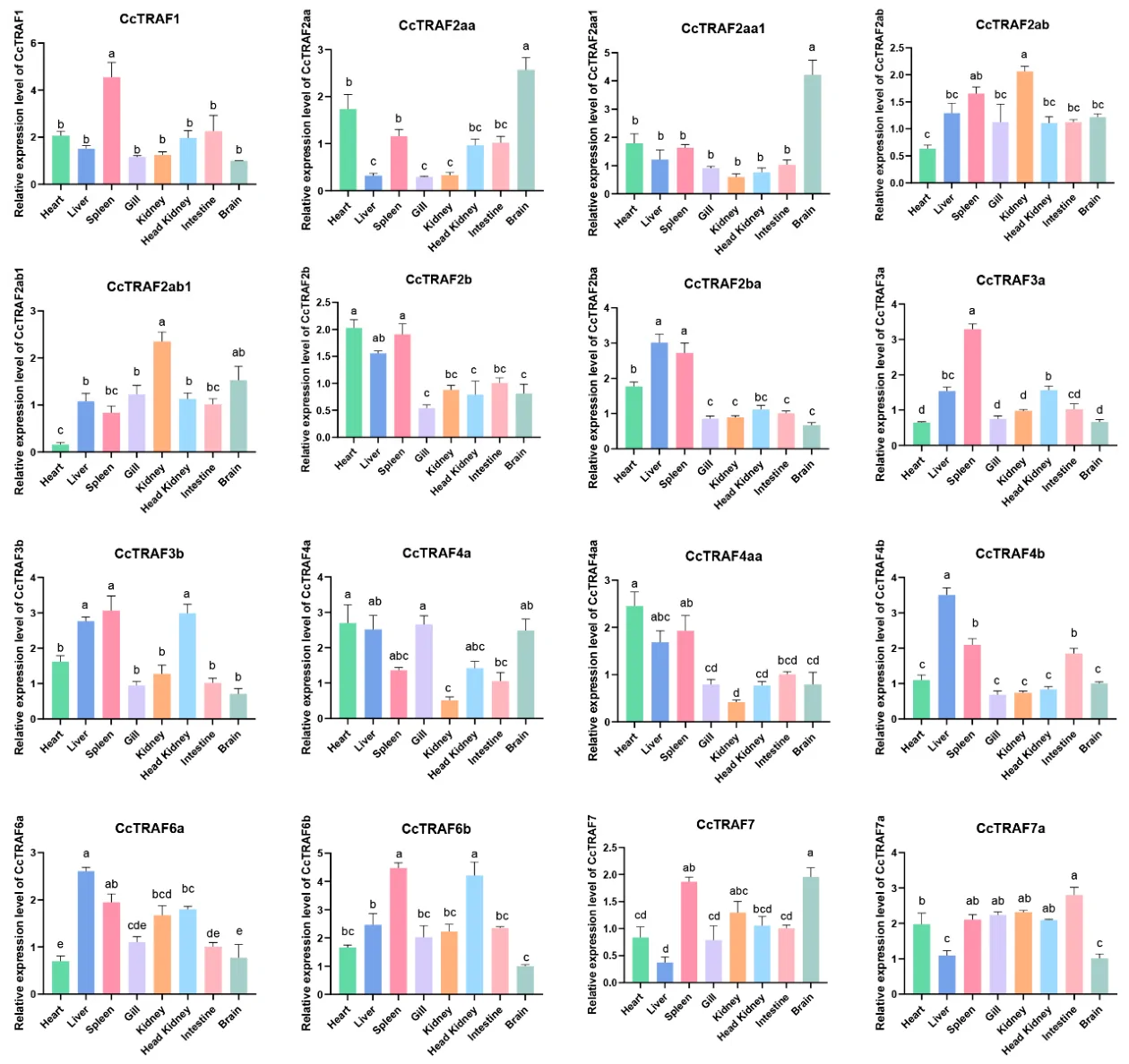
Relative expression levels of *CcTRAF* genes in tissues of healthy common carp. The data are represented as the mean ± standard deviation of three biological replicates. Each biological replicate represents tissue samples obtained from one individual fish originating from one independent experimental tank. Values with the same letter are not significantly different (*p* < 0.05).

To investigate the tissue distribution characteristics of the TRAF family in common carp, this study examined the mRNA expression levels of 16 *CcTRAF* genes across various tissues of healthy common carp. The results showed that all members of the *CcTRAF* family exhibited significant tissue-specific expression patterns, with different paralogs displaying distinct tissue expression preferences (Figure 6). Specifically, *CcTRAF1/2b/2ba/3a/6b/7a* were significantly enriched in the spleen; *CcTRAF2ba/4b/6a* were predominantly expressed in the liver; *CcTRAF2ab/2ab1* showed high expression in the kidney, while *CcTRAF3b/6a/6b* maintained elevated transcription levels in the head kidney; *CcTRAF4a/4aa* were highly expressed in the heart; and *CcTRAF2aa/2aa1/7* were primarily and specifically upregulated in the brain. Overall, the majority of *CcTRAF* genes were highly expressed in core immune organs such as the liver, spleen, and head kidney, whereas only a few paralogs showed dominant expression in the heart and brain.

### 3.8. Temporal Expression Patterns of CcTRAFs Under Viral Challenge

**Figure 7:**
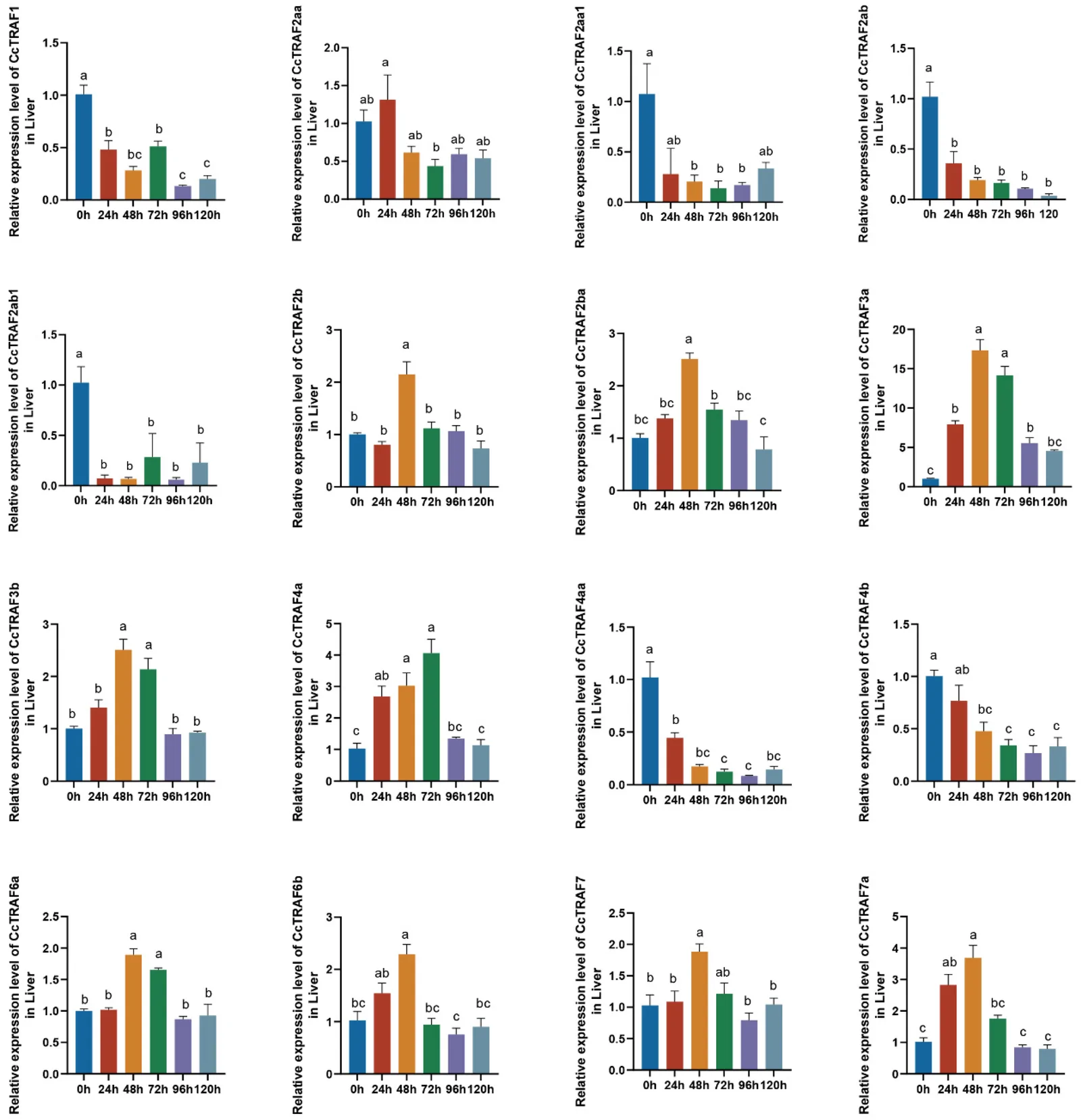
Temporal expression profiles of *CcTRAF* genes in the liver under CyHV-3 challenge. Expression values were normalized to the 0 h baseline. The data are presented as the mean ± standard deviation of three biological replicates. Each biological replicate represents tissue samples obtained from one individual fish originating from one independent experimental tank. Expression levels with different letters are significantly different (*p* < 0.05).

**Figure 8:**
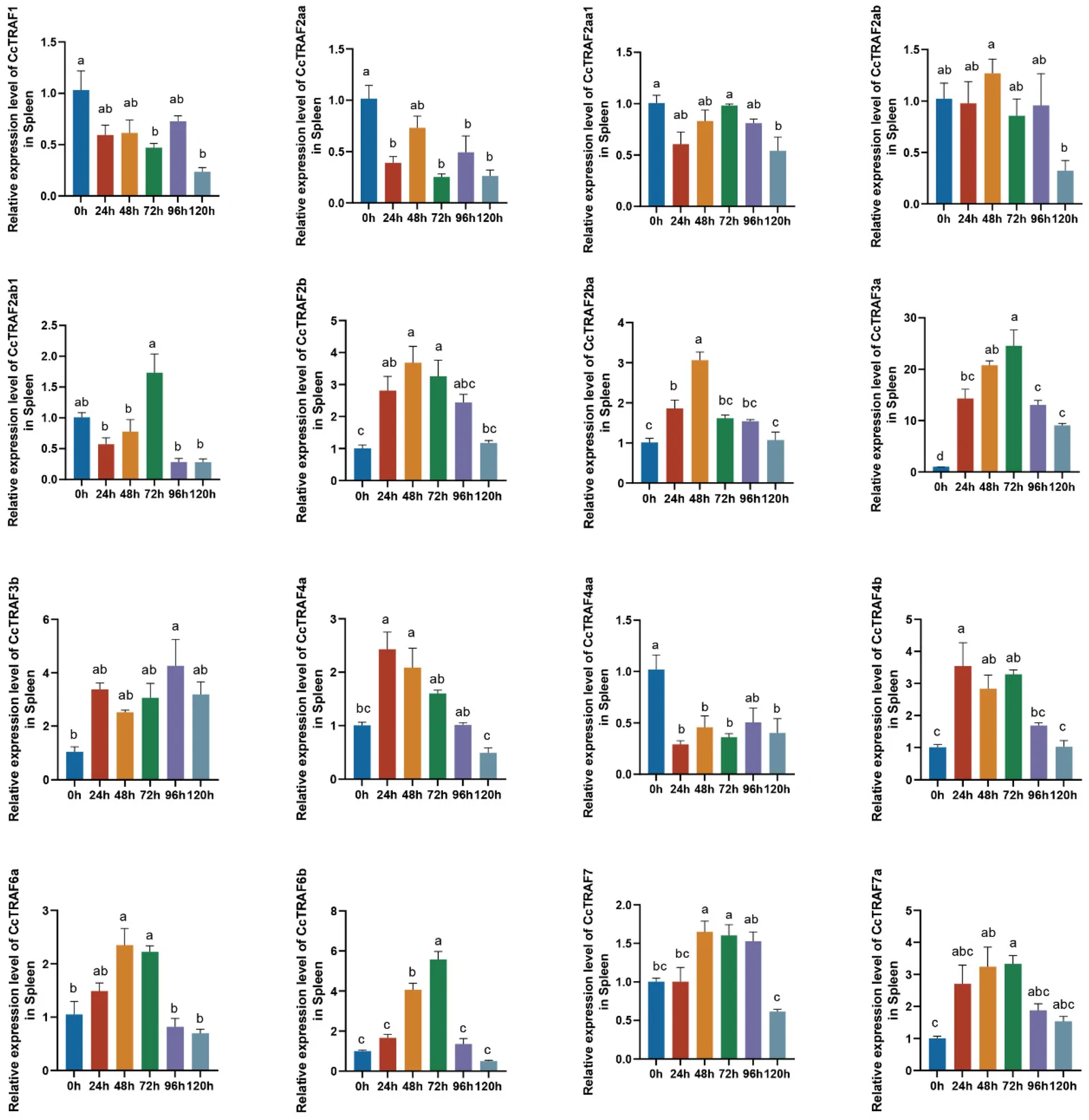
Temporal expression profiles of *CcTRAF* genes in the spleen under CyHV-3challenge. Expression values were normalized to the 0 h baseline. The data are presented as the mean ± standard deviation of three biological replicates. Each biological replicate represents tissue samples obtained from one individual fish originating from one independent experimental tank. Expression levels with different letters are significantly different (*p* < 0.05).

**Figure 9:**
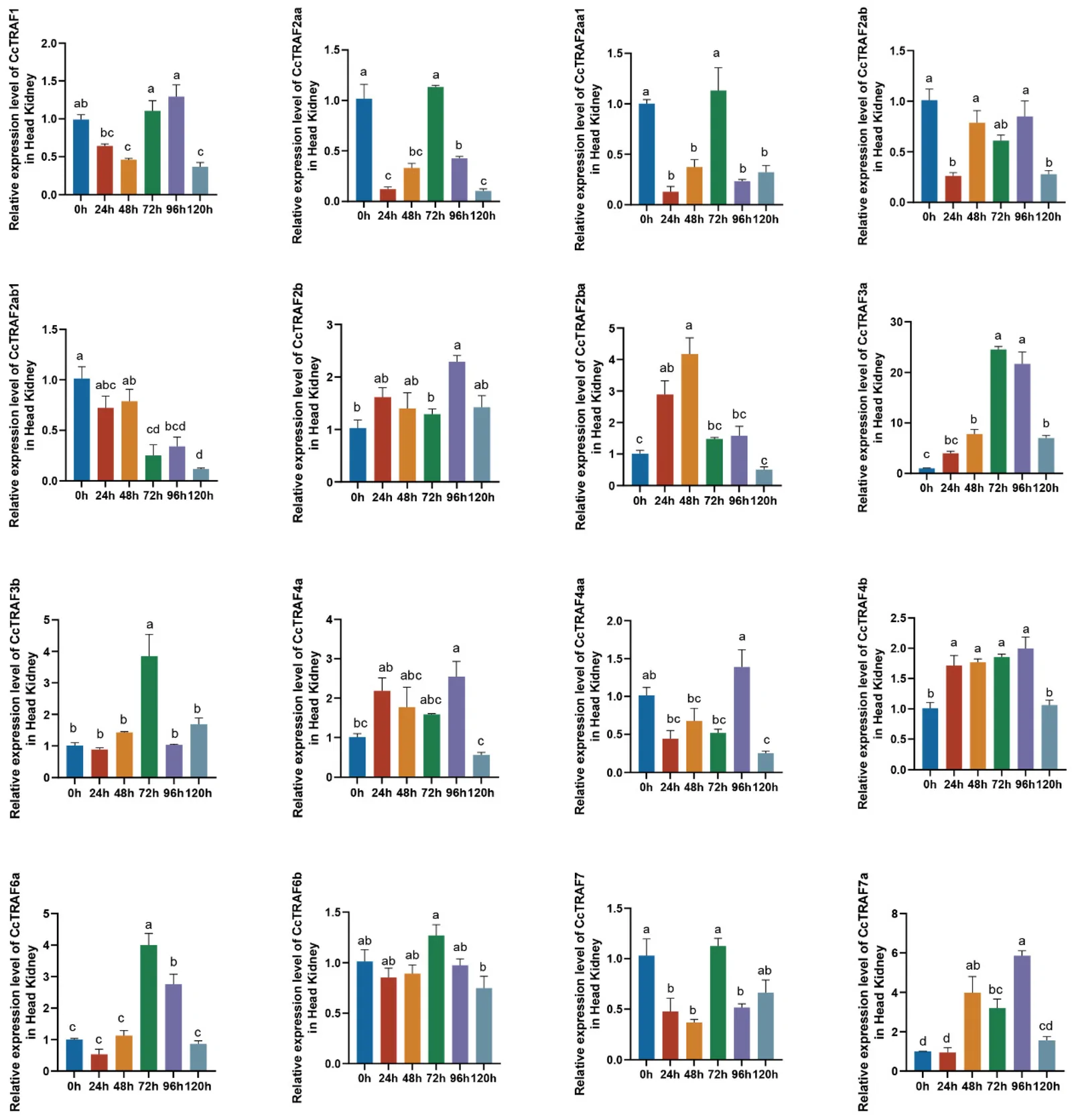
Temporal expression profiles of *CcTRAF* genes in the head kidney under CyHV-3 challenge. Expression values were normalized to the 0 h baseline. The data are presented as the mean ± standard deviation of three biological replicates. Each biological replicate represents tissue samples obtained from one individual fish originating from one independent experimental tank. Expression levels with different letters are significantly different (*p* < 0.05).

To explore the potential immune roles of *CcTRAF* genes during CyHV-3 challenge, the temporal mRNA expression profiles of *CcTRAFs* in the liver, spleen and head kidney were analyzed at 0, 24, 48, 72, 96 and 120 h post-challenge. The 0 h samples collected from challenged tanks were used as the baseline for temporal expression comparison. In the liver, *CcTRAF2b*/*2ba*/*3a*/*3b*/*6a*/*6b*/*7*/*7a* exhibited significant upregulation at 48 h post-challenge. All of these genes peaked at this time point and subsequently declined, showing highly consistent expression profiles. *CcTRAF4a* reached its highest expression level at 72 h, whereas other detected genes displayed different degrees of downregulation during the later challenge stages (Figure 7). In the spleen, *CcTRAF2b*/*2ba* presented similar expression trends to those in the liver, with the highest expression level occurring at 48 h. The transcripts of *CcTRAF3a*/*3b*/*6a*/*6b*/*7*/*7a* were significantly upregulated and peaked at 72 h, while the remaining genes were downregulated to varying extents (Figure 8). In the head kidney, *CcTRAF1*/*2aa*/*2aa1/2ab*/*2ab1*/*4aa*/*7* showed a unified dynamic expression pattern characterized by initial downregulation, subsequent upregulation, and sustained decrease in the late challenge stage. In contrast, the temporal expression patterns of *CcTRAF2b*/*2ba*/*3a*/*3b*/*6a*/*6b*/*7a* were consistent with their profiles in the liver and spleen (Figure 9).

## 4. Discussion

*TRAF*, as a key class of intracellular signal transduction proteins, plays an important role in vital biological processes such as immune regulation and inflammatory responses. This study conducted a whole-genome identification of *TRAF* gene family members in common carp, analyzed their evolutionary relationships and expression patterns, providing theoretical foundations for a deeper understanding of the immune regulatory mechanisms in common carp. Phylogenetic analysis divided TRAF proteins of common carp into six subfamilies (*TRAF1/2/3/4/6/7*). *TRAF1* and *TRAF2* clustered tightly and likely derived from one ancestral gene, consistent with their functional redundancy documented in mammals [21]. In contrast, *TRAF7* formed an isolated clade distant from other subfamilies, a topology possibly linked to its roles in regulating non-canonical NF-κB and other signaling cascades [22]. All seven TRAF members are single-copy in humans and mice, reflecting high evolutionary conservation of this gene repertoire in mammals [23]. Teleost TRAF families display obvious lineage-specific differentiation relative to mammals: *TRAF2* is widely expanded to 2–3 copies, whereas *TRAF1* and *TRAF5* are frequently lost across teleost lineages. Consistently, turbot, snakehead, fourfinger threadfin, humpback grouper and brown-marbled grouper carry three *TRAF2* paralogs, and yellow catfish harbors two *TRAF2* copies; these lines of evidence confirm *TRAF2* expansion as a universal evolutionary trait in teleosts [24,25,26,27,28,29]. We identified one *TRAF1* homolog and no *TRAF5* in common carp, matching the typical TRAF gene set of teleosts as seen in zebrafish. Nevertheless, common carp exhibited a unique TRAF duplication pattern distinct from all reported teleost species. While only *TRAF2* duplicates in other teleosts and the rest of the TRAF genes remain single-copy, the entire common carp TRAF family underwent massive expansion. Common carp contains six *TRAF2* paralogs (*TRAF2aa/2aa1/2ab/2ab1/2b/2ba*), and *TRAF3*, *TRAF4*, *TRAF6* and *TRAF7* each possess 2–3 duplicated copies. This genome-wide extensive duplication differentiates carp from other teleosts, which can be explained by lineage-specific variations in ancestral whole-genome duplication (WGD). Vertebrates underwent two rounds of ancient WGD events; teleosts experienced an additional teleost-specific third WGD, while allopolyploid cyprinids such as common carp have evolved via a distinctive fourth WGD [30]. We propose that repeated WGD events combined with allopolyploidization collectively trigger large-scale expansion and obvious subfunctionalization of *CcTRAF* paralogs. Abundant immune paralogs generated during these polyploidization processes equip carp with an immune system more complex and diversified than those of mammals and other teleosts [31].

Conserved motif and domain analyses revealed conserved structural features and distinct evolutionary divergence of the *CcTRAF* family. Most *CcTRAF* proteins exhibited highly consistent motif distribution, whereas *CcTRAF7* contained only three conserved motifs, indicating high intra-family sequence conservation. Domain prediction showed that nearly all CcTRAFs possessed canonical RING, zf-TRAF, and MATH domains, which are essential for core TRAF functions. Distinctively, *CcTRAF7* contained seven tandem WD40 domains at the C-terminus, consistent with the structural characteristics of mammalian TRAF7 [32]. The unique BIRC3 domain present only in *CcTRAF1/2* is involved in anti-apoptotic regulation, suggesting functional specificity of these two paralogs [33,34]. Intraspecific synteny analysis identified multiple segmental duplication pairs in the *CcTRAF* family, indicating that segmental duplication serves as a major driving force for *CcTRAF* expansion. This duplication and retention pattern is consistent with that of other teleosts after whole-genome duplication, such as zebrafish [35].

The expression profiles of TRAF family genes vary markedly across different teleost species and tissues. In the present study, all *CcTRAF* genes were ubiquitously detected in all examined tissues of common carp, while distinct differences were observed in the transcriptional abundance and tissue distribution patterns of individual family members. Furthermore, most *CcTRAF* genes exhibited preferential high expression in immune-related tissues. Among them, *CcTRAF1*, *CcTRAF2b*, *CcTRAF2ba*, *CcTRAF3a*, *CcTRAF6b* and *CcTRAF7a* were significantly highly expressed in the spleen. As the primary peripheral immune organ of fish, the spleen is enriched with abundant lymphocytes and phagocytes, acting as a critical immune tissue for eliminating invading pathogens and triggering systemic immune responses [36,37]. Consistent expression patterns have also been verified in previous research on TRAF genes of the brown-marbled grouper (*Epinephelus fuscoguttatus*) [28]. In addition, *CcTRAF3b*, *CcTRAF6a* and *CcTRAF6b* displayed relatively high transcriptional levels in the head kidney. The head kidney is a unique central immune organ exclusive to teleosts. It not only synthesizes and secretes diverse immune cytokines, but also serves as the primary site for lymphocyte differentiation and maturation, playing a vital regulatory role in immune responses against pathogenic invasion [38,39]. Consistent with our present findings, previous studies have reported that TRAF genes of snakehead fish (*Channa argus*) and other species also display preferentially high expression in the head kidney [25]. Meanwhile, several *CcTRAF* members, including *CcTRAF2ba*, *CcTRAF4b* and *CcTRAF6a*, also maintained high transcriptional levels in the liver. The liver possesses dual metabolic and immune functions, which can rapidly recognize invading pathogens and activate innate immune signaling pathways [40,41,42]. Identical results were also obtained from the tissue expression analysis of the TRAF family in fourfinger threadfin (*Eleutheronema tetradactylum*) [26]. In addition, *CcTRAF2aa*, *CcTRAF2aa1* and *CcTRAF7* exhibited tissue-specific upregulation in brain tissue. Collectively, members of the *CcTRAF* family displayed divergent expression patterns across various tissues, indicating that individual *CcTRAF* genes may perform diverse biological functions in common carp.

To explore the immunoregulatory functions of *CcTRAF* family genes during antiviral responses, the present study detected transcriptional expression changes in *CcTRAF* members in the liver, spleen and head kidney under CyHV-3 challenge. Members of the TRAF family exert divergent regulatory functions during immune responses. Among them, TRAF1 and TRAF4 are well-characterized negative immune regulators in vertebrates. They suppress excessive activation of TLR and IL-17 signaling pathways via distinct molecular mechanisms, jointly maintaining inflammatory homeostasis in the organism [43,44]. After yellow catfish (*Pelteobagrus fulvidraco*) were infected with *Edwardsiella ictaluri*, the transcript levels of *PfTRAF1* were significantly downregulated in the head kidney and gill. *PfTRAF4a* exhibited synchronous low expression in the head kidney, spleen, gill and brain, whereas *PfTRAF4b* showed a tissue-specific response: its expression was decreased only in the head kidney and spleen, while it was upregulated in the brain [29]. The present study revealed that the overall expression of *CcTRAF1* and *CcTRAF4* was suppressed in all immune tissues of common carp under CyHV-3 challenge. Our transcript level observations suggest a potential negative-feedback pattern mediated by *TRAF1* and *TRAF4*, which may contribute to alleviating excessive inflammatory responses and limiting tissue immune damage.

As a classic adaptor protein, TRAF2 mediates the activation of NF-κB and JNK signaling cascades to initiate host anti-pathogen immune responses [45]. After orange-spotted grouper (*Epinephelus coioides*) were infected with *Cryptocaryon irritans*, *EcTRAF2* exhibited significantly elevated expression in the gill and head kidney at most time points analyzed. Further in vitro cell assays verified that both *EcTRAF2-1* and *EcTRAF2-2* markedly enhanced NF-κB transcriptional activity, demonstrating that TRAF2 counteracts pathogenic stress by triggering downstream inflammatory pathways [46]. In the present study, distinct response patterns to CyHV-3 challenge were observed among various *CcTRAF2* paralogs: *CcTRAF2aa*, *CcTRAF2aa1*, *CcTRAF2ab* and *CcTRAF2ab1* were significantly downregulated, whereas *CcTRAF2b* and *CcTRAF2ba* were dramatically upregulated. These transcriptional responses imply that different *TRAF2* paralogs may have divergent potential roles in NF-κB and JNK-related signaling, possibly participating in balancing the magnitude of host inflammatory signals.

TRAF3 acts as a central adaptor molecule in the RLR/TLR signaling axis. It can recruit TBK1 to activate the IRF3/7 pathway for the production of type I interferons, and also exerts bidirectional regulatory effects on NF-κB signaling activity [47]. The expression of *LcTRAF3* was markedly upregulated in the gills of large yellow croaker under pathogenic bacterial stimulation. In Japanese sea bass infected with nervous necrosis virus, *LjTRAF3* served as an upstream molecule to fully initiate interferon-dependent antiviral programs [47,48]. Consistent with the patterns observed in the above species, *CcTRAF3* exhibited sustained high expression in the liver, spleen and head kidney of common carp at 48 h and 72 h post CyHV-3 challenge, revealing its long-term involvement in host antiviral immune defense.

TRAF6 is a critical E3 ubiquitin ligase for the TLR, NLR and IL-17 pathways. It mediates K63-linked ubiquitination modification of TAK1 to continuously trigger downstream NF-κB and MAPK inflammatory cascades [49]. Upon *Edwardsiella ictaluri* infection, splenic PfTRAF6 exhibited altered expression profiles and participated in regulating immune responses as well as facilitating the release of multiple pro-inflammatory cytokines in yellow catfish [29]. Upon bacterial infection, sustained high expression of OnTRAF6 was detected in the head kidney and blood of Nile tilapia [50]. Results of the present study demonstrated that *CcTRAF6* expression was significantly elevated in the liver and spleen of common carp at 48 h post CyHV-3 challenge, showing prominent transcriptional induction during the early transcriptional response under CyHV-3 challenge.

TRAF7 binds to MEKK3 to modulate NF-κB and p38 pathways. In teleosts, this molecule synergistically executes antibacterial and antiviral immune functions by concurrently activating NF-κB and IRF3/7 signaling [16]. After large yellow croaker were treated with the viral mimic poly I:C, *LcTRAF7* was significantly induced in the gills, head kidney and spleen, which implicates its conserved antiviral function [16]. In this study, the expression of *CcTRAF7* exhibited varying upregulation under CyHV-3 challenge in all immune tissues of common carp, and its expression peak gradually declined over time. This trend is highly consistent with the expression patterns reported in previous studies, implying that CcTRAF7 represents a candidate gene associated with the transcriptional immune response upon CyHV-3 challenge. Further functional validation, including gene manipulation, protein-level detection and viral replication assays, will be required to dissect the exact biological roles of these CcTRAF paralogs.

Despite these findings, several limitations of this study should be noted. In this experimental design, serial tissue sampling at 24, 48, 72, 96 and 120 h was performed only for the CyHV-3-challenged groups, with no parallel sampling of the PBS control group at these post-challenge time points. As feeding was stopped following immersion challenge, we could not fully distinguish transcriptional changes induced by CyHV-3 from time-dependent physiological shifts, handling stress, and fasting effects. Furthermore, the viral inoculum was prepared from tissue homogenate of diseased carp, whereas the control tanks received only PBS without a matrix-matched sham control. Non-viral constituents in the tissue homogenate may partly account for the observed transcriptional variation. Therefore, the temporal gene expression profiles reported here reflect transcriptomic dynamics under CyHV-3 challenge relative to the pre-challenge baseline (0 h in challenge tanks), instead of specific effects directly elicited by CyHV-3. Additional experiments with time-series sampling in sham/PBS control groups and matrix-matched mock inoculation are needed to decouple virus-specific immune responses from other confounding physiological factors.

## 5. Conclusions

In summary, the present study systematically performed genome-wide identification, evolutionary analysis, tissue expression profiling and expression profiling under CyHV-3 challenge of the *CcTRAF* gene family in common carp. Tissue expression profiles revealed that all *CcTRAF* genes were ubiquitously distributed across all tested tissues, with preferentially high expression in immune organs including the spleen, head kidney and liver. Under viral challenge, different CcTRAF genes displayed varied expression responses. *CcTRAF3/6/7* represent candidate genes associated with the transcriptional antiviral response under CyHV-3 challenge in common carp. The above findings clarify the differential response characteristics of the TRAF family in the innate immunity of common carp against CyHV-3, supplement the research data on TRAF gene functions in teleost fish, and lay a foundation for further elucidation of the antiviral molecular mechanisms and breeding of disease-resistant common carp.

## Figures and Tables

**Table 1:** Primer sequence of qRT-PCR.

Gene	ID	Forward Primer	Reverse Primer
*CcTRAF1*	XM_042729437.1	GCATCTGCGATGAAGTCCCT	CACCGTTTGACGCGCTTTAT
*CcTRAF2aa*	XM_042724010.1	CAGGAACCCTCACCACCATCTT	CACAGATGCTTGTCCTCCAGTT
*CcTRAF2aa1*	XM_042755485.1	CGTGGTACGCACTTGTCTCT	TGGTCCAGTAACATGAGGGTG
*CcTRAF2ab*	XM_042723211.1	GGTCATCAAAAACATGTCGCTCA	GCTCGTGATCGTGGATCTTCT
*CcTRAF2ab1*	XM_042752123.1	CAACCGGACTGTTGGATCAGG	CAGCCTGGTTTGAGGATTGAC
*CcTRAF2b*	XM_042756907.1	ATTGAAGTGGGTCAACATGAAGG	GCTCATTGTGGCGGTCTTTC
*CcTRAF2ba*	XM_042725057.1	AGCCGTACTGTCCTCTGTGC	CCGCGATACAATCCCAGTCC
*CcTRAF3a*	XM_042774568.1	CGGCTTCAGGGACCGATTT	TTGTCGAGGGTTACAGAGCAC
*CcTRAF3b*	XM_042742456.1	CACCGAGTTACAGAAACACAAG	TGGAAGAAAAGGAGCAGTGATT
*CcTRAF4a*	XM_042739259.1	CCGGCGAGTACGACAACCT	CAGCGAAGGGTCGCTCTG
*CcTRAF4aa*	XM_042771029.1	GGCGAGTACGACAGCCTC	ATGTTGAGGTTTGGACAGCGA
*CcTRAF4b*	XM_042711146.1	GAAGTCGAGATGGAACACTGC	CACACTCCCAGAAGTCTGCCT
*CcTRAF6a*	XM_042760650.1	GTCACTCAGTAGCTGCCCA	TACACTGGCATGACTCTGGC
*CcTRAF6b*	XM_042728184.1	ACAGAACACCGGACCGAAAC	GTCGCTGTCACAGTAGGGAT
*CcTRAF7*	XM_042722803.1	TCACGCTTCGATCGTTTCTCA	TTTGGTCCGGTTGCTGATGT
*CcTRAF7a*	XM_042719944.1	ATTACCCACAATCCTCTGGCAT	ATTGGTGCTGTATCAAAGTCTGC
*β-actin*	M24113.1	GCCGTGACCTGACTGACTACCT	GCCACATAGAGAGCTTCTCCTTG

**Table 2:** Identification of the *TRAF* gene family members and prediction results of the physicochemical properties of their encoded proteins.

	Gene ID	ORF	AA	MW	PI	II	AI	GRAVY
*CcTRAF1*	109051965	1659	552	61,612.70	6.73	45.51	77.86	−0.455
*CcTRAF2aa*	109063188	1725	574	65,054.34	8.21	48.98	80.57	−0.358
*CcTRAF2aa1*	109063179	1728	575	65,153.36	8.39	48.54	79.76	−0.383
*CcTRAF2ab*	109066138	1752	583	65,573.38	8.24	48.01	75.45	−0.404
*CcTRAF2ab1*	109103466	1761	586	65,983.04	8.32	46.88	76.23	−0.404
*CcTRAF2b*	109088491	1512	503	56,339.90	8.55	44.81	77.61	−0.354
*CcTRAF2ba*	109065003	1611	536	60,199.91	8.53	45.86	74.46	−0.426
*CcTRAF3a*	109051440	1641	546	62,560.32	7.21	45.11	73.50	−0.425
*CcTRAF3b*	109071136	1722	573	65,589.64	8.07	48.26	71.10	−0.487
*CcTRAF4a*	109077348	1413	470	54,416.35	8.33	51.74	63.26	−0.633
*CcTRAF4aa*	109104220	1413	470	54,395.36	8.33	52.25	64.30	−0.606
*CcTRAF4b*	109083222	1440	479	54,832.72	8.04	49.33	72.84	−0.517
*CcTRAF6a*	109065463	1632	543	61,773.50	5.88	54.48	75.40	−0.451
*CcTRAF6b*	109093401	1710	569	64,739.85	6.29	51.75	74.18	−0.437
*CcTRAF7*	109050251	2022	673	74,982.37	6.56	41.11	80.19	−0.271
*CcTRAF7a*	109064531	2178	725	81,142.81	6.94	40.71	84.39	−0.214

ORF: open reading frame; AA: amino acids; MW: Molecular weight; II: instability index; AI: Aliphatic index; GRAVY: Grand average of hydropathicity.

## Data Availability

The original contributions presented in this study are included in the article. Further inquiries can be directed to the corresponding author.

## Supplementary Materials

The following supporting information can be downloaded at: https://www.mdpi.com/article/10.3390/ani16182859/s1; Figure S1: Tertiary structure of encoded proteins of the CcTRAF gene family members; Table S1: Information of TRAF family genes used in this study; Table S2: Secondary structures of encoded proteins of CcTRAF; Table S3: Quality evaluation of homologous modeling for CcTRAF.
