# Supplementary material for: Genome-Wide Identification of the TRAF Gene Family in Common Carp (Cyprinus carpio) and Analysis of Their Expression in Response to CyHV-3 Challenge
Source: Animals (Basel). 2026 Sep 11;16(18):2859. doi: 10.3390/ani16182859 (PMC13603144; doi:10.3390/ani16182859)
Supplement: Supplementary file 1 [file animals-16-02859-s001.zip › animals-4510215-supplementary.pdf]

Supplementary Material

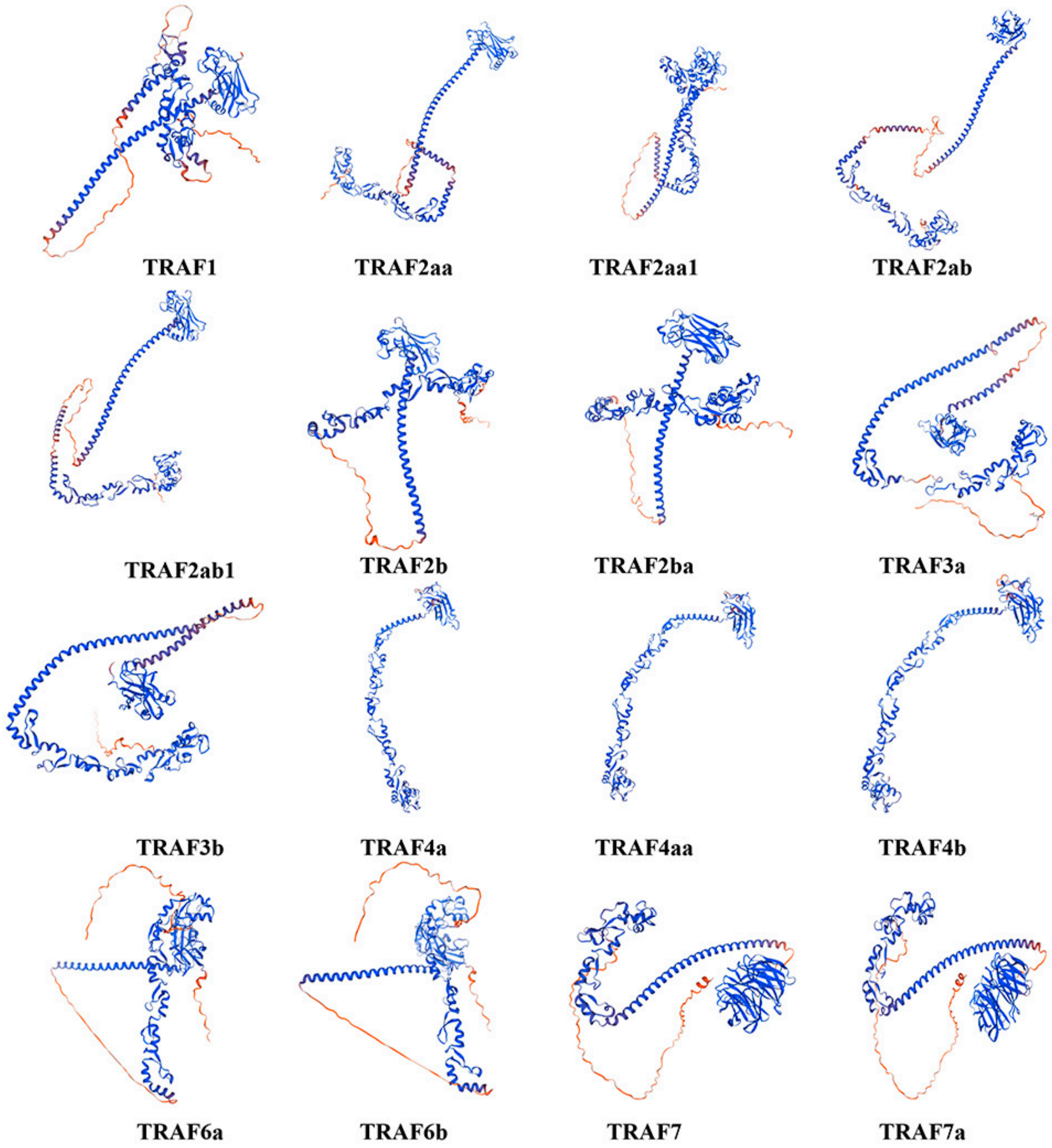

Supplementary Figure S1. Tertiary structure of encoded proteins of the *CcTRAF* gene family members.

Supplementary Table S1. Information of TRAF family genes used in this study.

No.	Species name	Gene ID	Accession ID
1	*Scophthalmus maximus*	*SmTRAF2aa*	XP_035464741.1
		SmTRAF2ab	XP_035494741.1
		SmTRAF2b	XP_035464621.1
		SmTRAF3	XP_035466077.1
		SmTRAF4a	XP_035478637.1
		SmTRAF5	XP_035493832.1
		SmTRAF6	XP_035483854.1
		SmTRAF7	XP_035470591.1
2	*Cynoglossus semilaevis*	*CsTRAF2aa*	XP_008323996.1
		CsTRAF2ab	XP_008334135.1
		CsTRAF2b	XP_008324226.1
		CsTRAF3	XP_008313252.1
		CsTRAF4	XP_008307618.1
		CsTRAF5	XP_016890423.1
		CsTRAF6	XP_008308373.1
		CsTRAF7	XP_024920211.1
3	*Paralichthys olivaceus*	*PoTRAF2aa*	XP_019949664.1
		PoTRAF2ab	XP_019949666.1
		PoTRAF2b	XP_019937630.1
		PoTRAF3	XP_019962478.1
		PoTRAF4	XP_019944060.1
		PoTRAF6	XP_019955056.1
		PoTRAF7	XP_019967403.1
4	*Oreochromis niloticus*	*OnTRAF2aa*	XP_005454516.1
		OnTRAF2ab	ENSONIT00000013084.2
		OnTRAF2b	ENSONIT00000003602.1
		OnTRAF3	ENSONIT00000014849.1
		OnTRAF4a	ENSONIT00000016577.1
		OnTRAF5	ENSONIT00000020328.1
		OnTRAF6	ENSONIT00000007404.1
		OnTRAF7	ENSONIT00000008289.1
5	*Takifugu rubripes*	*TrTRAF2aa*	ENSTRUT00000015701.1
		TrTRAF2ab	XP_011617711.2
		TrTRAF2b	ENSTRUT00000047965.1
		TrTRAF3	ENSTRUT00000013410.1
		TrTRAF4a	ENSTRUT00000012939.1
		TrTRAF6	ENSTRUT00000041841.1
		TrTRAF7	ENSTRUT00000034208.1
6	*Gasterosteus aculeatus*	*GaTRAF2aa*	ENSGACT00000006992.1
		GaTRAF2ab	XP_040051981.1
		GaTRAF2b	ENSGACT00000022124.1
		GaTRAF3	ENSGACT00000005719.1
		GaTRAF4a	ENSGACT00000014787.1
		GaTRAF5	ENSGACT00000025934.1
		GaTRAF6	ENSGACT00000022691.1
		GaTRAF7	ENSGACT00000007697.1
7	*Oryzias latipes*	*OlTRAF2aa*	ENSORLT00000020861.1
		OlTRAF2ab	XP_020561925.1
		OlTRAF2b	ENSORLT00000008990.1
		OlTRAF3	ENSORLT00000024258.1
		OlTRAF4a	ENSORLT00000005580.1
		OlTRAF5	ENSORLT00000001369.1
		OlTRAF6	ENSORLT00000001291.1
		OlTRAF7	ENSORLT00000020808.1

8	*Lepisosteus oculatus*	*LoTRAF1*	ENSLOCT00000001629.1
		LoTRAF2a	ENSLOCT00000007756.1
		LoTRAF2b	ENSLOCT00000000945.1
		LoTRAF3	ENSLOCT00000012036.1
		LoTRAF4a	ENSLOCT00000007097.1
		LoTRAF5	ENSLOCT00000019207.1
		LoTRAF6	ENSLOCT00000001266.1
		LoTRAF7	ENSLOCT00000006911.1
9	*Xiphophorus maculatus*	*XmTRAF2ab*	ENSXMAT00000004927.1
		XmTRAF2b	ENSXMAT00000005534.1
		XmTRAF3	ENSXMAT00000018475.1
		XmTRAF4a	ENSXMAT00000011914.1
		XmTRAF6	ENSXMAT00000011865.1
		XmTRAF7	ENSXMAT00000008769.1
10	*Danio rerio*	*DrTRAF1*	ENSDART00000101174.6
		DrTRAF2a	ENSDART00000043466.7
		DrTRAF2b	ENSDART00000165672.2
		DrTRAF3	ENSDART00000031458.8
		DrTRAF4a	ENSDART00000016024.9
		DrTRAF4b	ENSDART00000056878.4
		DrTRAF5	ENSDART00000040550.8
		DrTRAF6	ENSDART00000038273.6
		DrTRAF7	ENSDART00000132703.3
11	*Mus musculus*	*MmTRAF1*	NP_001313530.1
		MmTRAF2a	NP_001277342.1
		MmTRAF3	NP_035762.2
		MmTRAF4	NP_033449.2
		MmTRAF5	NP_035763.2
		MmTRAF6	NP_001290202.1
		MmTRAF7	NP_001165584.1
12	*Homo sapiens*	*HsTRAF1*	NP_001177874.1
		HsTRAF2	NP_066961.2
		HsTRAF3	NP_003291.2
		HsTRAF4	NP_004286.2
		HsTRAF5	NP_001306136.1
		HsTRAF6	NP_004611.1
		HsTRAF7	NP_115647.2

Supplementary Table S2. Secondary structures of encoded proteins of CcTRAF

	α-Helix (%)	β-Turn (%)	Extended strand (%)	Random coil (%)
CcTRAF1	45.11	3.62	10.51	40.76
CcTRAF2aa	45.99	3.31	10.28	40.42
CcTRAF2aa1	46.43	2.96	9.74	40.87
CcTRAF2ab	43.40	3.60	10.81	42.20
CcTRAF2ab1	49.32	3.75	9.22	37.71
CcTRAF2b	46.52	3.78	12.33	37.38
CcTRAF2ba	46.46	3.73	12.31	37.50
CcTRAF3a	42.49	3.11	11.72	42.67
CcTRAF3b	42.93	3.84	11.17	42.06
CcTRAF4a	38.94	2.55	14.26	44.26
CcTRAF4aa	36.17	2.55	13.19	48.09
CcTRAF4b	37.37	3.13	12.53	46.97
CcTRAF6a	41.25	2.58	10.31	45.86
CcTRAF6b	37.26	2.64	12.65	47.45
CcTRAF7	26.30	5.50	24.96	43.24
CcTRAF7a	30.21	5.10	24.00	40.69

Supplementary Table S3. Quality evaluation of homologous modeling for CcTRAF

Protein name	Template ID	Sequence identity (%)	Sequence coverage (%)	Modelled residues	Model quality metric (GMQE)	Sequence similarity with template (%)
CcTRAF1	B0UXK1	84.57	100	1-552	0.81	57
CcTRAF2aa	E7EXH5	98.43	100	1-574	0.87	62
CcTRAF2aa1	E7EXH5	97.21	100	1-575	0.87	61
CcTRAF2ab	A0A5A9P7Z1	91.25	100	1-583	0.83	59
CcTRAF2ab1	A0A1A7YF87	88.87	100	1-586	0.83	59
CcTRAF2b	A0A286YAJ9	91.98	99	1-503	0.84	59
CcTRAF2ba	A0A286YAJ9	92.67	99	1-536	0.86	60
CcTRAF3a	A0A6P7HDF6	78.82	99	1-546	0.84	55
CcTRAF3b	Q6DEG0	93.02	100	1-573	0.86	60
CcTRAF4a	Q7T1K2	98.94	100	1-470	0.92	63
CcTRAF4aa	Q7T1K2	98.72	100	1-470	0.92	63
CcTRAF4b	Q7T1K2	74.20	98	1-477	0.91	55
CcTRAF6a	Q6IWL4	88.75	100	1-543	0.81	59
CcTRAF6b	Q6IWL4	88.56	95	27-569	0.78	59
CcTRAF7	A0A2R8QGD3	98.29	96	29-673	0.78	62
CcTRAF7a	A0A2R8QGD3	98.29	89	81-725	0.73	62
